# Probiotic-mediated p38 MAPK immune signaling prolongs the survival of *Caenorhabditis elegans* exposed to pathogenic bacteria

**DOI:** 10.1038/s41598-021-00698-5

**Published:** 2021-10-28

**Authors:** Miroslav Dinić, Stefan Jakovljević, Jelena Đokić, Nikola Popović, Dušan Radojević, Ivana Strahinić, Nataša Golić

**Affiliations:** Laboratory for Molecular Microbiology (LMM), Institute of Molecular Genetics and Genetic Engineering (IMGGE), University of Belgrade, Belgrade, Serbia

**Keywords:** Immunology, Microbiology

## Abstract

The host-microbiota cross-talk represents an important factor contributing to innate immune response and host resistance during infection. It has been shown that probiotic lactobacilli exhibit the ability to modulate innate immunity and enhance pathogen elimination. Here we showed that heat-inactivated probiotic strain *Lactobacillus curvatus* BGMK2-41 stimulates immune response and resistance of the *Caenorhabditis elegans* against *Staphylococcus aureus* and *Pseudomonas aeruginosa*. By employing qRT-PCR and western blot analysis we showed that heat-inactivated BGMK2-41 activated PMK-1/p38 MAPK immunity pathway which prolongs the survival of *C. elegans* exposed to pathogenic bacteria in nematode killing assays. The *C. elegans pmk-1* mutant was used to demonstrate a mechanistic basis for the antimicrobial potential of BGMK2-41, showing that BGMK2-41 upregulated PMK-1/p38 MAPK dependent transcription of C-type lectins, lysozymes and tight junction protein CLC-1. Overall, this study suggests that PMK-1/p38 MAPK‐dependent immune regulation by BGMK2-41 is essential for probiotic-mediated *C. elegans* protection against gram-positive and gram-negative bacteria and could be further explored for development of probiotics with the potential to increase resistance of the host towards pathogens.

## Introduction

Innate immunity provides the first line of defense against invading microbes with nonspecific detection of conserved features of pathogens^1^. In mammals, discrimination of these conserved molecules occurs through Pattern Recognition Receptors (PRRs), such as Toll-like Receptors (TLRs) resulting in activation of intracellular signaling cascades that rapidly induce the expression of distinct genes involved in the inflammatory and immune responses^2^. The molecular pathways initially triggered by pathogens are highly conserved in a large variety of organisms ranging from flies and nematodes to mammals^3^.

*Caenorhabditis (C.) elegans* is a small soil nematode naturally exposed to numerous microbes. From early adulthood, nematodes feed on bacteria which pass through the pharynx to the gut, colonize the intestinal lumen and establish their gut microbiota^4^. This close interaction of nematode and bacteria led *C. elegans* to evolve ways of avoiding dangerous pathogens and to adapt to hostile environment^5^. To defend against infection, *C. elegans* possesses certain evolutionarily conserved defense mechanisms including the PMK-1 pathway, an orthologue of p38 mitogen-activated protein kinase (MAPK)^6^, the transforming growth factor β (TGF-β) signaling denote as DBL-1 pathway^7^ and the ERK1/2 MAPK homolog MPK-1 that can be activated in response to specific pathogens^5^. The most studied PMK-1 pathway coordinates defense against a variety of ingested pathogens by regulating the expression of different antimicrobial effectors. Downstream targets of PMK-1 include genes that encode antimicrobial proteins, such as antimicrobial peptides, C-type lectins and lysozymes^3^. Like in mammals, pathogen recognition in worms could be mediated through TLR ortholog TOL-1, which is required for immunity of *C. elegans* to certain gram-negative bacteria^8^. As most bacterial species that are known to be pathogenic for *C. elegans* also cause infections in humans^9^, *C. elegans* has become a useful model system for innate immunity studies in terms of pathogen-host interactions^10^.

In laboratory conditions, *C. elegans* are reared on a single bacterial strain of *Escherichia coli* OP50^4^. The composition of bacterial diet plays an important role in the control of *C. elegans* well-being by modulating animal physiology including metabolism, development and reproduction^11^. These bacterivore nematodes have been used to study dietary effects and changes in physiology and transcriptomic signatures after exposure to commensal bacteria and probiotics^12^. Probiotics are recognized as beneficial microorganisms, known to exert numerous positive effects on human health^13,14^. Probiotic-host cross-talk primarily results in the modulation of innate immunity which is important to determine the host resistance to infection^15^. Several studies already demonstrated that some *Lactobacillus* strains when applied alive activated immune and stress responses in the worms which increased resistance of the nematodes to certain bacterial infections^16,17^. Beside live probiotic bacteria, UV‐killed and heat-inactivated bacteria, also exhibited beneficial effects on the worms. It has been shown that lactobacilli applied unviable, as postbiotics, tune SKN-1/NRF2 or HLH-30/TFEB signaling in the nematodes which consequently extended *C. elegans* lifespan and improved general health markers^18,19^. However, the data about potential of heat-inactivated probiotic bacteria to enhance survival of the host towards different pathogens, especially *Staphylococcus aureus* and *Pseudomonas aeruginosa,* remain limited. Reports showed that *S. aureus* and *P. aeruginosa* infection causes death of the *C. elegans* by triggering intestinal epithelium distention and destruction followed by intracellular invasion and complete degradation of internal organs^20^. This means that epithelial barrier represents the major site of host defense system, beside above-mentioned canonical pathways^21^. In light of the growing antibiotic resistance of these two pathogens, the stimulation of innate immunity and maintenance of barrier integrity, as alternative strategy, is increasingly being explored.

Therefore, following the postbiotic trend of replacing live probiotic bacteria with bacterial biomolecules and/or inactivated bacteria in order to get stable and better controlled biological response^22^, here we report that heat-inactivated *Lactobacillus curvatus* BGMK2-41 strain activated PMK-1/p38 MAPK dependent immune response to control the production of hosts antimicrobials which increased survival of the worms exposed to both gram-positive and gram-negative pathogenic bacteria.

## Results

### Pre-conditioning of *C. elegans* with BGMK2-41 prolongs the survival of nematodes exposed to pathogenic bacteria

**Figure 1:**
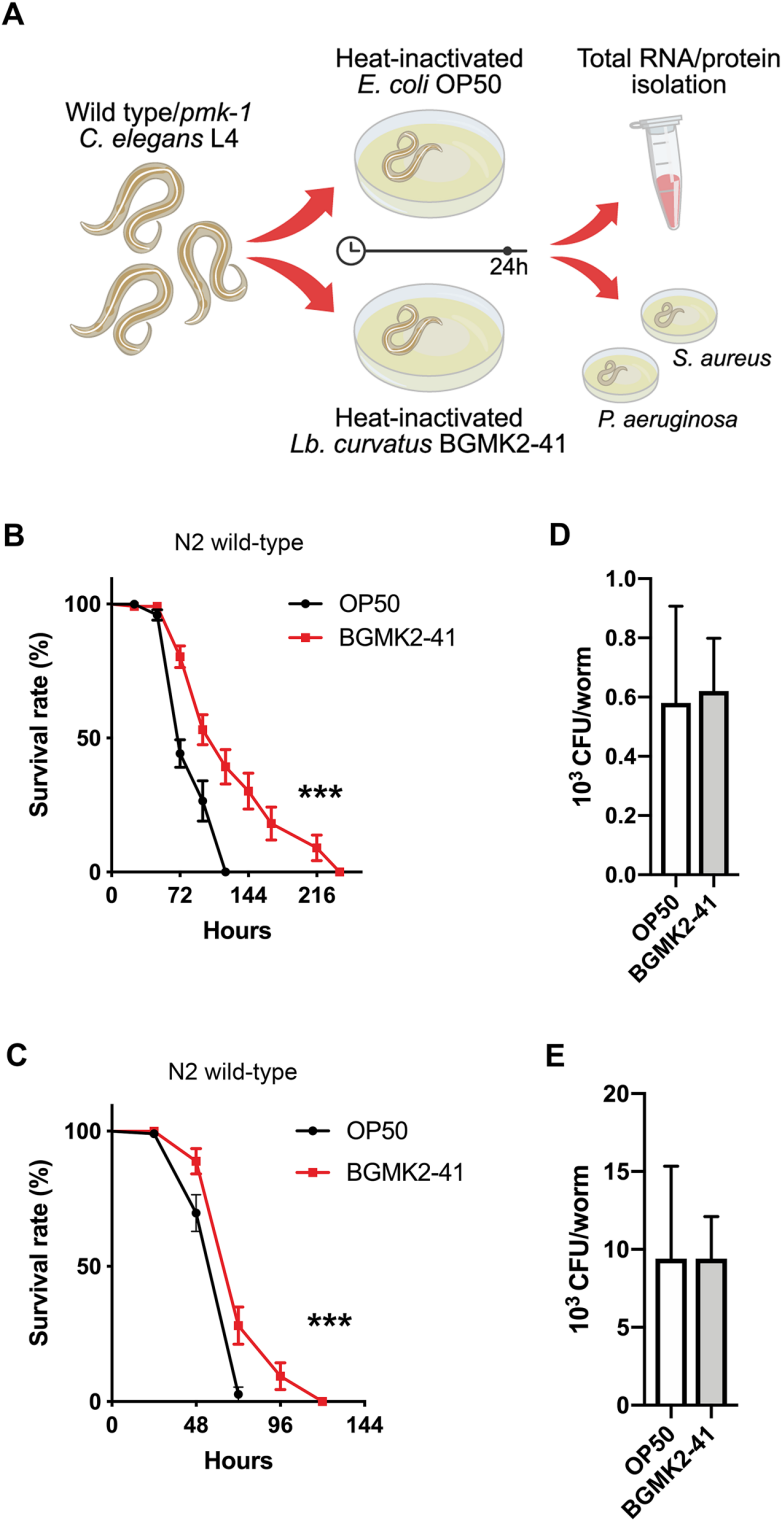
Heat-inactivated *Lactobacillus curvatus* BGMK2-41 prolongs the survival of *C. elegans* exposed to pathogenic bacteria. (**A**) An overview of experimental approach for evaluation of BGMK2-41 effects on infected worms’ lifespan and gene/protein. Survival curve of WT animals exposed to (**B**) *S. aureus* ATCC 25923 and (**C**) *P. aeruginosa* PA14 after overnight feeding of L4 stage worms with heat-inactivated control (*E. coli* OP50) or BGMK2-41 bacteria (n = 25–30 worms per group, results are representative of 2 independent assays). CFUs level of (**D**) *S. aureus* ATCC 25923 and (**E**) *P. aeruginosa* PA14 present in the BGMK2-41 pre-treated worms after 1 day of exposure to pathogens (n = 10 worms per group, results are representative of 2 independent assays). All values are presented as mean ± SD and the student’s t-test was used to compare the treated group relative to control. The log-rank (Mantel-Cox) test was used to assess the p-value in nematode killing assays (***P < 0.001). The statistical analysis and graphs were done in GraphPad Prism version 8.0.0 for Mac, GraphPad Software, www.graphpad.com. (**A**) was designed by using free trial of Adobe Illustrator, www.adobe.com/products/illustrator.

To evaluate the immunomodulatory potential of heat-inactivated BGMK2-41 we used *C. elegans* as an in vivo model system and schematic overview of experimental approach is presented in Fig. 1A. Results revealed that animals previously exposed to heat-inactivated BGMK2-41 exhibited enhanced survival upon infection with both *S. aureus* ATCC 25923 (Fig. 1B) and *P. aeruginosa* PA14 (Fig. 1C) pathogenic strains in comparation to the exposure to heat-inactivated OP50, used as a control. Furthermore, we were able to demonstrate that gram-negative bacteria exhibit more lethal infection which correlates with the increased intestinal accumulation of *P. aeruginosa* PA14 (9.4 × 10^3^ CFUs), whereas only 0.58 × 10^3^ CFUs accumulated in the case of *S. aureus* ATCC 25923. However, prolonged survival of nematodes previously exposed to BGMK2-41 was not a consequence of decreased intestinal load of pathogenic bacteria, as we detected no differences in CFUs between BGMK2-41 treated and control animals (Fig. 1D,E). These findings imply that BGMK2-41 enhances the survival of *C. elegans* without decreasing bacterial accumulation and colonization.

### Heat-inactivated BGMK2-41 activates a canonical PMK-1/p38 MAPK immune pathway

**Figure 2:**
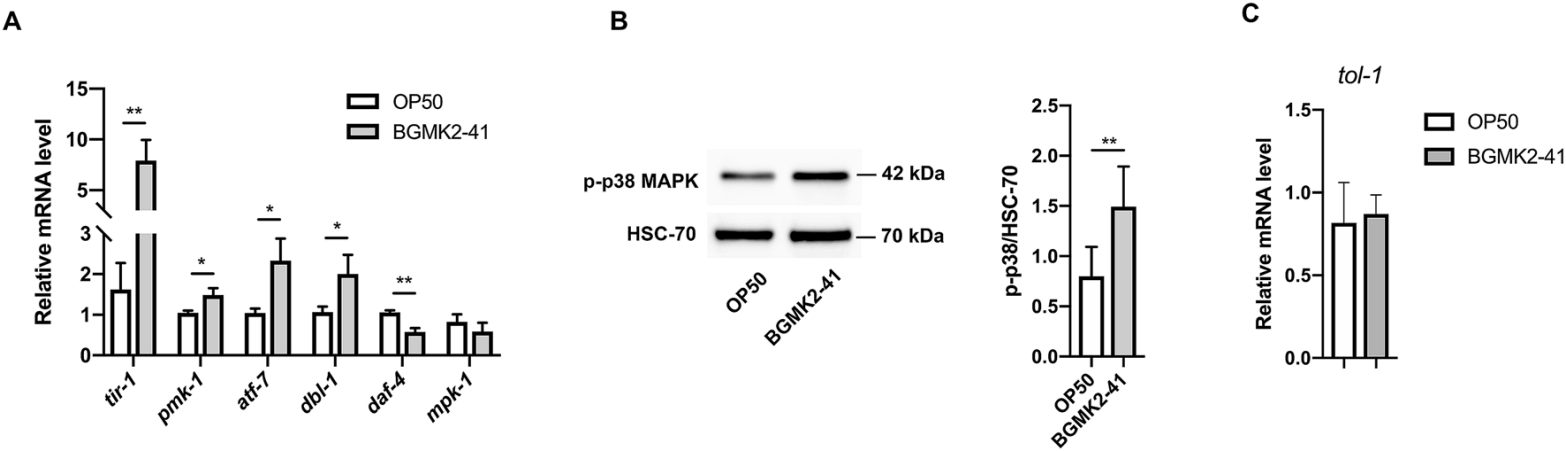
Heat-inactivated *Lactobacillus curvatus* BGMK2-41 stimulates a canonical p38MAPK immune pathway in *C. elegans.* (**A**) Expression of immune-related genes measured by qRT-PCR in WT animals after 24 h of treatment with heat-inactivated BGMK2-41 relative to heat-inactivated *E. coli* OP50 control. (**B**) Representative western blot with densitometric analysis showing the levels of phospho-p38 MAPK isolated from WT animals after overnight BGMK2-41 treatment. HSC-70 was used as a loading control. (**C**) Levels of *tol-1* mRNA measured in day 1 old WT worms treated with BGMK2-41. All results were obtained from three independent experiments and the data are presented as mean ± SD and student’s t-test was used to compare the treated group relative to control (*P < 0.05, **P < 0.01). The statistical analysis and graphs were done in GraphPad Prism version 8.0.0 for Mac, GraphPad Software, www.graphpad.com.

To address whether BGMK2-41 upregulates gene expression of the above-mentioned immune pathways, we performed qRT-PCR analysis in wild-type N2 worms. We have found that all genes involved in PMK-1/p38 MAPK signaling (*tir-1*, *pmk-1*, *atf-7*) were significantly upregulated (Fig. 2A). Remarkably, we detected elevated mRNA levels for *dbl-1* gene, but when we analyzed further DBL-1 signaling, we detected decreased transcription of downstream *daf-4* gene (Fig. 2A). Finally, *mpk-1* expression was similar in BGMK2-41 treated and control worms which excluded ERK1/2 MAPK involvement in worms’ resistance phenomenon (Fig. 2A). Next, we confirmed the activation of PMK-1/p38 MAPK pathway by determining the level of the activated form of p38 MAPK and showed that its phosphorylation was upregulated in *C. elegans* treated with BGMK2-41 relative to OP50 control (Fig. 2B).

When we looked upstream of PMK-1/p38 MAPK pathway and examine evolutionarily conserved TOL-1/TLR role in immune response stimulated by BGMK2-41, we did not detect differences in *tol-1* mRNA level between treated and control groups (Fig. 2C). Therefore, we concluded that BGMK2-41 triggers a canonical PMK-1/p38 MAPK immune pathway, most likely in TOL-1/TLR independent manner and that alternative mechanisms may exist for the detection of conserved BGMK2-41 molecules.

### The PMK-1/p38 MAPK is essential for the enhanced resistance of the worms to pathogenic bacteria

**Figure 3:**
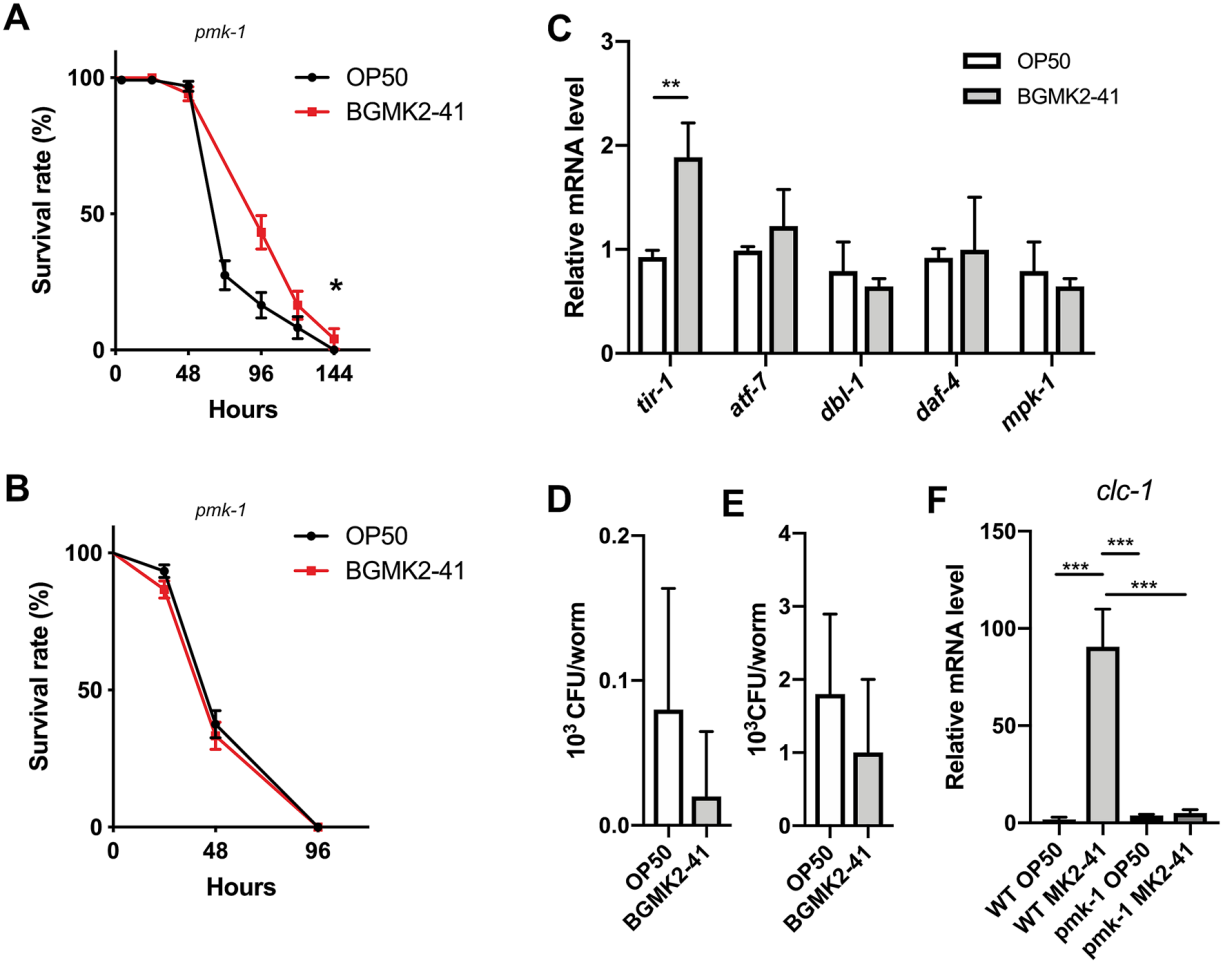
The p38 MAPK deficiency abrogates protective effects of *Lactobacillus curvatus* BGMK2-41. Survival curve of *pmk-1* loss-of-function animals exposed to (**A**) *S. aureus* ATCC 25923 and (**B**) *P. aeruginosa* PA14 after overnight feeding of L4 stage worms with heat-inactivated control (*E. coli* OP50) or BGMK2-41 bacteria (n = 25–30 worms per group, results are representative of 2 independent assays). (**C**) Expression of immune-related genes analyzed by qRT-PCR in the L4 stage of the *pmk-1* mutant after overnight treatment with heat-inactivated BGMK2-41 (n = 3, three independent experiments). CFUs values of (**D**) *S. aureus* ATCC 25923 and (**E**) *P. aeruginosa* PA14 present in the *pmk-1* deficient BGMK2-41 treated worms after 1 day of exposure to pathogens (n = 10 worms per group, results are representative of 2 independent assays). (**F**) Tight junction barrier assessment by determination of *clc-1* mRNA levels in day 1 old WT and *pmk-1* worms treated with heat-inactivated BGMK2-41 (results from three independent experiments). All values are presented as mean ± SD. Student’s t-test was used to compare the treated group relative to control. The log-rank (Mantel-Cox) test was used to assess the p-value in nematode killing assays (*P < 0.05, **P < 0.01, ***P < 0.001). The statistical analysis and graphs were done in GraphPad Prism version 8.0.0 for Mac, GraphPad Software, www.graphpad.com.

To evaluate whether PMK-1/p38 MAPK is functionally involved in host defense against pathogenic bacteria triggered by BGMK2-41, we infected *C. elegans* loss-of-function *pmk-1* mutant and followed survival over time. Deletion of *pmk-1* reduced enhanced survival induced by pre-conditioning of the worms with BGMK2-41 during exposure to *S. aureus* ATCC 25923 (Fig. 3A). Moreover, increased resistance of the worms previously exposed to BGMK2-41 was completely abrogated in *pmk-1* mutant exposed to *P. aeruginosa* PA14 (Fig. 3B). Inactivation of *pmk-1* did not substantially alter BGMK2-41 induced expression of *tir-1* mRNA, a gene encoding a highly conserved Toll/IL-1 resistance (TIR) domain protein acting upstream of PMK-1, observed in *pmk-1* mutant (Fig. 3C). On the other hand, mRNA levels of downstream *atf-7* transcription factor responsible for the expression of secreted immune effectors, were not changed upon treatment with BGMK2-41 suggesting that BGMK2-41 activates TIR-1, but impaired PMK-1/p38 MAPK signaling did not elicit protective response against pathogens (Fig. 3C). The defective PMK-1/p38 MAPK signaling was confirmed by the absence of p38 MAPK phosphorylation in *pmk-1* mutant (Supplementary Fig. 1). Finally, qRT-PCR analysis in *pmk-1* mutant showed that genes involved in DBL-1 and MPK-1 immune pathways were unchanged by BGMK2-41 treatment excluding the possible activation of these two defense programs (Fig. 3C).

Gut colonization analysis for both pathogens in *pmk-1* mutant, showed similar CFUs values between the experimental and control groups, as we previously determined in N2 worms (Fig. 3D,E). This observation excluded the impact of BGMK2-41 on pathogens gut colonization and confirmed that prolonged survival of BGMK2-41 treated worms was independent from pathogen intestinal burden.

As immune activation did not result in a decrease of pathogens in the intestines, we further looked in the integrity of the intestinal barrier. We tested the expression of mRNA encoding the tight junction protein claudin-like in *Caenorhabditis* (CLC-1), mainly expressed in the epithelial cells of digestive tubes^23^. The expression analysis revealed that BGMK2-41 strongly stimulated the expression of the claudin-related *clc-1* gene in N2 worms, but this effect was completely abolished in *pmk-1* mutant suggesting that BGMK2-41 strengthen gut epithelial barrier through upregulation of tight junction’s claudin in PMK-1/p38 MAPK dependent manner (Fig. 3F).

### The p38 MAPK pathway triggered by heat-inactivated BGMK2-41 controls expression of various antimicrobial genes

**Figure 4:**
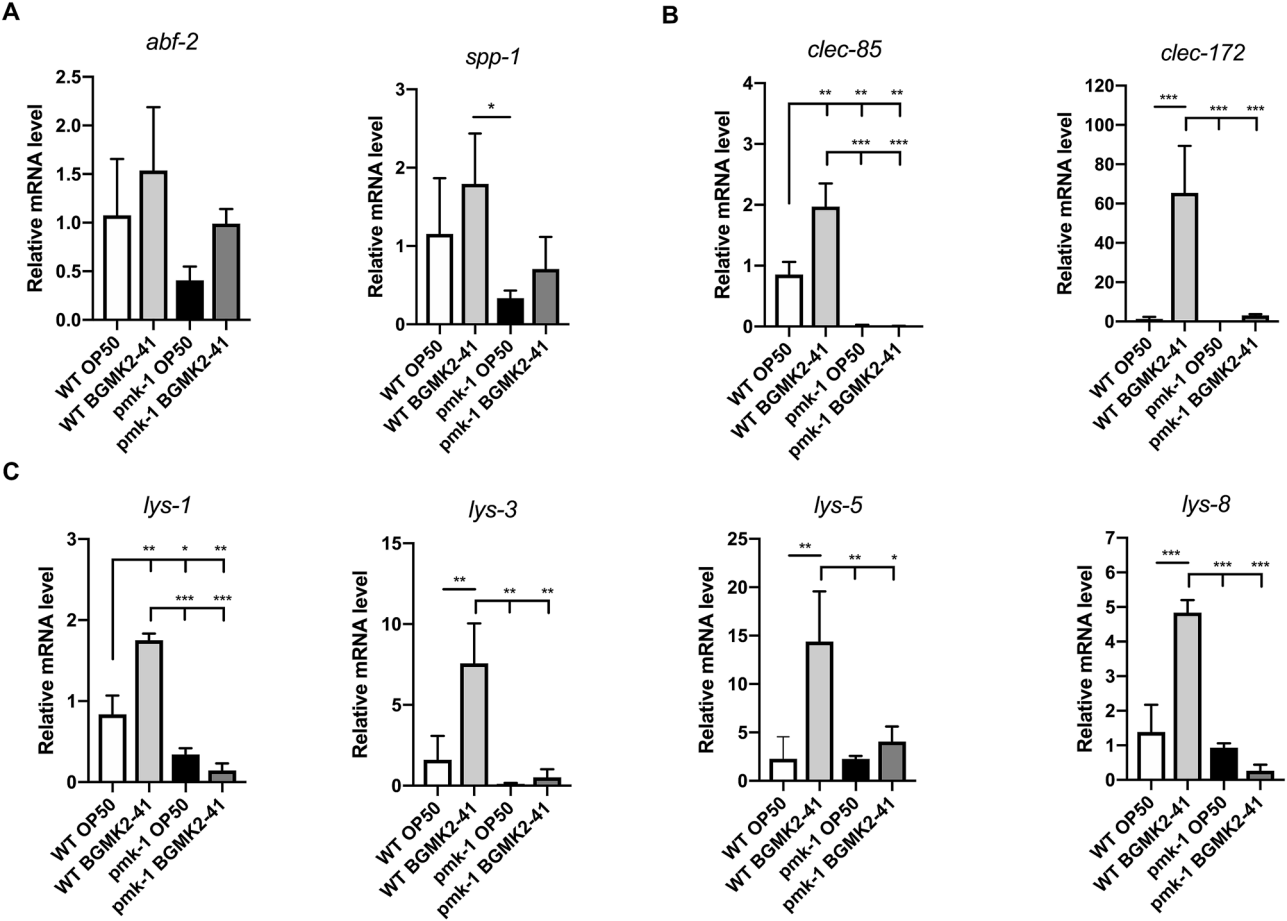
Heat-inactivated *Lactobacillus curvatus* BGMK2-41 controls the expression of antimicrobial proteins via p38 MAPK. Expression of (**A**) antimicrobial peptides, (**B**) C-type lectins and (**C**) lysozymes measured by qRT-PCR in day 1 old WT and *pmk-1* worms treated with heat-inactivated BGMK2-41 (results from three independent experiments). All values are presented as mean ± SD. Student’s t-test was used to compare the treated group relative to control (*P < 0.05, **P < 0.01, ***P < 0.001). The statistical analysis and graphs were done in GraphPad Prism version 8.0.0 for Mac, GraphPad Software, www.graphpad.com.

Next, we performed transcriptional profiling of the selected antimicrobial genes as markers of the wider host response, by comparing their expression in N2 animals and *pmk-1* mutants after exposure to BGMK2-41. These genes include antimicrobial peptides (*abf-2*, *spp-1*), C-type lectins (*clec-85*, *clec-172*) and lysozymes (*lys-1*, *lys-3*, *lys-5*, *lys-8*). First, the BGMK2-41 failed to upregulate the antimicrobial peptides both in N2 and *pmk-1* animals, excluding their role in BGMK2-41 orchestrated defense program (Fig. 4A). However, the BGMK2-41 treatment of N2 worms results in a major upregulation of the rest of the tested genes, with *clec-172* and *lys-5* as the most highly expressed (Fig. 4B,C). Remarkably, a drastic reduction of mRNAs of C-type lectins and lysozymes were detected in *pmk-1* worms (Fig. 4B,C). As a result, we concluded that BGMK2-41 enhanced survival of the worms is mediated by the production of C-type lectins and lysozymes as antimicrobial effectors controlled by PMK-1/p38 MAPK activity.

## Discussion

Probiotic lactobacilli can elicit distinct immunomodulatory effects in the host ranging from immune stimulation to suppression in a species-specific manner^24,25^. In the present study, we found that PMK-1/p38 MAPK‐dependent immune activation by heat-inactivated *Lb. curvatus* BGMK2-41 is essential for *C. elegans* protection against *S. aureus* ATCC 25923 and *P. aeruginosa* PA14*,* as loss of PMK-1/p38 MAPK almost completely abrogated survival of the worms exposed to pathogens. Further, we show that p38 MAPK orchestrated a specific transcriptional response which strengthened the gut epithelial barrier and stimulated the production of C-type lectins and lysozymes to improve the survival of infected worms.

Despite a growing body of evidence reporting the beneficial effects of different *Lactobacillus* species, only a few studies have attempted to elucidate the health-promoting potential of the *Lb. curvatus* strains*.* The literature data showed that different isolates of *Lb. curvatus* exhibit beneficial role in dyslipidemia and obesity^26^*.* Moreover, in the case of *Lb. curvatus* effects on the immune system, one report has shown that *Lb. curvatus* WiKim38 triggered the activation of dendritic cells and stimulated IL-10 production to suppress chemically-induced colitis in mice^27^. However, as the data about *Lb. curvatus* impact on gut epithelial barrier, mucosal and innate immunity is lacking, our study provides evidence of the immunostimulatory potential of *Lb. curvatus*.

The p38 MAPK has been established as a major regulator of the innate immune response, especially with regard to the production of proinflammatory cytokines^28^. Its modulation by probiotics, like *Lb. acidophilus* NCFM, has been described as a key factor in controlling the production of cytokines and chemokines of gut epithelial cells necessary for the restriction of pathogen invasion^29^. Like in mammals, PMK-1/p38 MAPK activation in *C. elegans* also mediates the expression of secreted immune response genes as a response to recognized microbe-associated molecular patterns (MAMPs)^3^. Beside pathogenic bacteria previously described to activate this pathway in nematodes, probiotic lactobacilli exhibit some of their beneficial effects through p38 MAPK. It has been described that *Lb. fermentum* JDFM216 effectively stimulated PMK-1/p38 MAPK pathway and increased longevity and immunity of *C. elegans* towards *S. aureus* and *E. coli*^30^. These results are in accordance with our findings in term of BGMK2-41 mediated protective effect against pathogens, but another study aimed to screen a library of different lactic acid bacteria (LAB) reported that the heat-inactivated LAB did not reduce the susceptibility of the worms to *P. aeruginosa*^31^. In this study, we detected the most prominent survival upon infection with gram-positive bacteria suggesting that BGMK2-41 and *S. aureus* share the same core network of host protective responses probably due to their structural similarities’ characteristic for gram-positive bacteria. To the best of our knowledge, this is the first study demonstrating the positive effect of heat-inactivated lactobacilli applied as postbiotic towards these two pathogens.

*S. aureus* and *P. aeruginosa* are frequent multiresistant nosocomial pathogens with increasing prevalence and ability to delay wound healing of hospitalized patients^32^. With growing resistance to antimicrobial agents, stimulation of the immune response by BGMK2-41 which enhanced resistance to *S. aureus* ATCC 25923 and *P. aeruginosa* PA14 infection, could be of great importance for patients affected by wound healing disorders and cutaneous infections^33^. Further, by applying the postbiotic concept, we succeeded to showed that heat-inactivated BGMK2-41 is sufficient to elicit an effective immune response against both pathogenic bacteria which excluded the potential adverse effect of live probiotics when it comes to their application to wounded tissue or intake by immunocompromise individuals^34^. Consistent with this, it has been shown that the gene expression profiles elicited by live or dead *En. faecium*, also used as probiotic^35^, are extremely similar and dependent on the immune regulator PMK-1/p38 MAPK^36^. Except on gene expression level, a protective effect of heat-inactivated *Lb. plantarum* 133 (LP133) and *Lb. fermentum* 21 (LP21) has been demonstrated against *Salmonella* and *Yersinia* infection in *C. elegans*^37^. These data pointed that conserved heat-stable moieties of *Enterococcus* and *Lactobacillus* mediated transcriptional response and worms’ survival to infection. It seems that BGMK2-41 with its heat-stable features evoked a protective response of the *C. elegans* against *S. aureus* ATCC 25923 and *P. aeruginosa* PA14. Additionally, failure of BGMK2-41 to activate TOL-1/TLR led to conclusion that potential other receptors are engaged in recognition of BGMK2-41 MAMPs, which is not surprise considering that a lot of studies pointed that the TOL-1/TLR signaling is not significantly involved in the primary response against gram-positive bacteria in the intestinal cells of *C. elegans*^38^. As TOL-1/TLR represents one of the major activators of antimicrobial peptide ABF-2, the unchanged expression of *abf-2* gene further justifies the conclusion that BGMK2-41 activates PMK-1/p38 MAPK in TOL-1/TLR independent fashion^8^. On the other hand, strong activation of TIR-1 observed in N2 worms and *pmk-1* mutant could control the innate immunity in *C. elegans* independently of TOL-1/TLR^39^. Although, the elevated mRNA levels for *dbl-1* gene pointed to the possible activation of this pathway as well, we excluded this possibility by detecting decreased expression of *daf-4* gene, which encodes receptor of TGF-beta signaling.

Probiotic lactobacilli and their secreted molecules have a well-described role in the reinforcement of the gut intestinal barrier by increasing expression of tight junction proteins^40,41^. As BGMK2-41-mediated *C. elegans* resistance to both tested pathogens was uncoupled from their CFUs number in the gut, we propose that upregulated expression of *clc-1* consequently strengthened the tight junctions, which may be responsible for the observed longevity extension of infected worms. Moreover, the important way that hosts can defend against the pathogens is by tolerating them^42^. A mechanism of tolerance does not result in decrease of pathogen load, which is in accordance with the results we obtained for BGMK2-41, suggesting that reinforcement of the gut intestinal barrier could occur due to increasing tolerance to infection. When it comes to p38 MAPK role in tight junction dynamics, we provide evidence that *clc-1* expression was dependent on p38 MAPK phosphorylation. It has been shown that p38 MAPK induced upregulation of claudin-1 during regeneration of rat hepatocytes^43^. On the contrary, p38 MAPK could mediate tight junction disruption in distal renal tubular cells induced by calcium oxalate crystals^44^. Therefore, having in mind the reported dual role of p38 MAPK, our study shows a significant positive correlation between p38 MAPK and claudin-like protein in in vivo model. Finally, lactobacilli could mediate worms’ resistance to methicillin-resistant *S. aureus* (MRSA) infection by employing DBL-1 immune signaling. This effect was independent from changes in MRSA gut colonization and CFUs^45^, which support our conclusion that BGMK2-41 does not have to affect the number of pathogenic bacteria to increase host survival during infection. Also, our CFUs result is in accordance with *S. aureus* and *P. aeruginosa* pathogenicity in the worms, which is reflected in pathogens accumulation in the intestinal lumen in the form of clumps surrounded by an extracellular matrix^20^. This characteristic phenotype of the infection could impact the CFUs numeration.

Taken together, our study offers a safer alternative besides the traditional use of antibiotics by providing molecular mechanism behind the immunostimulatory effect of heat-inactivated *Lb. curvatus* BGMK2-41. The results presented here indicate that modulation of the p38 MAPK pathway could be of particular interest in order to restrain infection via strengthening the gut epithelial barrier and innate immunity.

## Materials and methods

### *C. elegans* strains and maintenance

*C. elegans* was maintained on nematode growth medium (NGM) plates seeded with *E. coli* OP50 strain at 20 °C by using standard growing protocols^46^. To obtained synchronized population egg-bearing worms from mix population were collected from NGM plates by using M9 buffer. Eggs were extracted in the cleaning solution containing 0.5 M NaOH with 1% Na-hypochlorite and then washed with M9 buffer at least three times. Eggs were transferred to OP50 seeded NGM plates and incubated overnight at 20 °C to obtain synchronized L1 animals. In order to avoid progeny hatching and maintain synchronized population, NGM plates were supplemented with 20 μM 5-Fluorodeoxyuridine (FudR, Sigma-Aldrich) where was necessary. The wild‐type N2 (Bristol) and KU25 *pmk-1* (km25) strains were used in the study. *E. coli* OP50 and worms’ strains originate from the collection of Caenorhabditis Genetics Center, University of Minnesota.

### Bacterial strains and growth conditions

Strain *Lactobacillus curvatus* BGMK2-41 is a part of the IMGGE collection of microorganisms previously isolated from an artisanal Serbian fermented dairy product Kajmak^47^. The strain was cultivated in MRS broth (Merck) at 37 °C under anaerobic conditions using Anaerocult A (Merck). *P. aeruginosa* PA14, *S. aureus* ATCC 25923 and *E. coli* OP50 were cultivated in LB broth at 37 °C with shaking/aerobic conditions.

### Preparation of heat-inactivated bacteria and treatments

Overnight grown culture of *Lb. curvatus* BGMK2-41 were pelleted by centrifugation at 5000 × g for 10 min at room temperature, washed twice in phosphate-buffered saline (PBS) and resuspended in the same volume of LB medium as OP50. Heat-inactivation for both BGMK2-41 and OP50 was performed at 60 °C for 60 min followed by inoculation of bacterial suspension (10 μl) on MRS/LB agar plates and incubation overnight at 37 °C in order to check the efficacy of inactivation treatment. The NGM plates were prepared by spreading the heat-inactivated bacterial suspensions on 9 cm plates and dried at room temperature. Age‐synchronous worms were grown to the L4 stage on live *E. coli* OP50 followed by transfer to NGM plates containing appropriate heat-inactivated treatment.

### Nematode killing assays

In the nematode killing assays, we used *S. aureus* ATCC 25923 and *P. aeruginosa* PA14 found to elicit distinct immune responses in the infected nematodes^20^. The L4 stage worms were preconditioned with heat-inactivated BGMK2-41 or OP50 for 24 h before being transferred to pathogens containing plates.

*P. aeruginosa* killing assay was done as described by Troemel et al.^3^ Briefly, overnight culture of *P. aeruginosa* PA14 strain was spread on modified 3.5 mm NGM plates containing 0.35% peptone and incubated for 24 h at 37 °C followed by additional incubation of the plates for two days at 25 °C. A total of 25–30 one day old adult worms were transferred to each pathogen plate and a total of 100–120 worms (4 plates) were used per condition.

*S. aureus* killing assay was performed by spreading the overnight culture of *S. aureus* ATCC 25923 strain onto 3.5 mm NGM plates and incubate for 24 h at 37 °C followed by transfer of 100–120 one day old adult worms to 4 pathogen plates per condition. All killing assays were performed at 20 °C and death events scored every day by gentle prodding with a platinum wire. The worms that escaped were censored.

### Colony forming unit assay (CFU)

The colonization of worms’ intestines with pathogenic bacteria was determined according to the method described by Yuen and Ausubel^36^, with slight modifications. Briefly, after the treatment with heat-inactivated BGMK2-41 or OP50, the worms were exposed to *S. aureus* ATCC 25923 and *P. aeruginosa* PA14 for 24 h. The infected nematodes were picked onto unseeded NGM agar plates in order to remove any external bacteria and then 10 worms were transferred to a 2 ml tube containing 25 mM tetramisole hydrochloride (Sigma-Aldrich) and 0.01% Triton X-100 (Sigma-Aldrich) in M9 wash buffer. The infected worms were washed 3 times with a total volume of 250 µl of the buffer. Afterwards, 200 µl of the buffer was removed and 100 µl of M9 buffer containing 20 mM tetramisole hydrochloride and 2% Triton X-100 and 0.5 mm silicon beads were added to the tubes. The tubes were vortexed in Disruptor Genie (Scientific Industries Inc, USA) at maximum speed for 5 min to release the bacteria from the worm intestine without impairing bacterial viability. The suspension was serially diluted and plated onto LB agar plates to enumerate the bacterial colonies. The results were expressed as CFU unit per worm.

### RNA isolation and quantitative real-time PCR (qRT-PCR)

Approximately 200 worms were collected from 9 cm treatment plates using M9 buffer and washed three times to remove the remaining bacteria. Total RNA was extracted using a Trizol reagent (Thermo Fisher Scientific) and treated with DNase I (DNA-free DNA Removal Kit) according to the manufacturer′s protocol (Thermo Fisher Scientific). RevertAid Reverse Transcriptase (Thermo Fisher Scientific) was used to transcribe 0.5 µg of isolated RNA as a template, with random hexamers (Thermo Fisher Scientific) and RiboLock RNase inhibitor (Thermo Fisher Scientific) also used in the reactions. Synthesized cDNA was then subjected to qRT-PCR analysis using SYBR Green PCR Master Mix (Thermo Fisher Scientific) in a 7500 real-time PCR machine (Applied Biosystems) under the following conditions: 10 min at 95 °C activation, 40 cycles of 15 s at 95 °C and 60 s at 60 °C. All results are normalized against the control *act-1* gene^19^ and expressed as relative target abundance using the 2^−ΔΔCt^ method^48^. Primers used in the study are listed in Table 1. All primers were purchased from Thermo Fisher Scientific. For each condition, three independent replicates were used.

**Table 1:** The list of primers used in the study.

Primer name	Primer sequence 5ʹ–3ʹ	Reference
*tir-1* forward	CCGACCACCAAAGAAATGCC	This work
*tir-1* reverse	CTTGGTCCACCGATGCTTCT	
*pmk-1* forward	ACTTCATCCGACTCCACGAG	This work
*pmk-1* reverse	CAGCAGCACAAACAGTTCCA	
*atf-7* forward	AGAAATAAGGCTGCGGCTGT	This work
*atf-7* reverse	TTTCAGCCTCCATTGCTTGA	
*tol-1* forward	GCTCACCAACATCGAGCAATTC	This work
*tol-1* reverse	GCGTTTCCAGCCAAATTCAC	
*clc-1* forward	CCACTCACCCTCTTTGCAGT	This work
*clc-1* reverse	CGAGTATCCAAGCTGCGAGT	
*mpk-1* forward	TGGAGGGCAGAATCCTGTTT	This work
*mpk-1* reverse	CAACAAGCTTTTCAGCGGGA	
*dbl-1* forward	TTTTGCGGCGAACAAATCGT	This work
*dbl-1* reverse	TTCGCTGTTGCCTGTTTGTG	
*daf-4* forward	GCCAAGGACGATCATTTCGC	This work
*daf-4* reverse	TGGGAACTTGTCCTTCTTCAA	
*act-1* forward	TGCAGAAGGAAATCACCGCT	^19^
*act-1* reverse	CGGACTCGTCGTATTCTTGC	
*abf-2* forward	TTCCTTGCACTTCTCCTGGC	This work
*abf-2* reverse	GACGACCGCTTCGTTTCTTG	
*spp-1* forward	TCGTCGAGGGTGGAGAGAAG	This work
*spp-1* reverse	ACGCCTTGTCTGGAGAATCC	
*clec-85* forward	ACTATGTCGCTGAGAGCACG	This work
*clec-85* reverse	TCCGGAGACTGGGTAAGACA	
*clec-172* forward	AGTTTGGGAATTCGTGCTACA	This work
*clec-172* reverse	CCGTGCTCCATTCCGAAGAT	
*lys-1* forward	GGATCTGGAGCATTCGACACA	This work
*lys-1* reverse	GCTGGGGAGGTAACCTGAATC	
*lys-3* forward	TGCGAAGAATCTGGGCTTGT	This work
*lys-3* reverse	GTGGCCTGTCTCCATGGTC	
*lys-5* forward	TGCAGTGTTGTAAGTGCAGC	This work
*lys-5* reverse	CATCGACATCAGTGAGGCCA	
*lys-8* forward	TTGTCCGTGCATACAACCCA	This work
*lys-8* reverse	TCCTTGCTTGCTTGAAGCCG	

### Western blotting

After worm’s collection (approximately 200 animals) from treatment plates, proteins were isolated according to the protocol described by Herholz et al.^49^ Protein concentration was measured with the BCA Assay Kit (Thermo Fisher Scientific) and 20 μg of extracted proteins were separated on 12% SDS–PAGE and transferred to a 0.2 mm nitrocellulose membrane (GE Healthcare). Western blotting was performed overnight at 4 °C with antibodies against: HSC-70 (1:2000, Santa Cruz Biotechnology, #sc-7298) and phospho-p38 MAPK (1:1000, Cell Signaling, #9211). Chemiluminescence was detected by using ChemiDoc Touch Imaging System with Image Lab Touch Software (Bio-Rad). The Image Lab Touch software accurately estimates the shortest exposure time. The Image Resolution/Sensitivity scale was set to 4 × 4 pixel binning settings and pictures captured by using Rapid Auto Exposure mode. The intensity of the bands was quantified in ImageJ (National Institutes of Health, NIH) software by using plot lanes and wand tool. The results were normalized to HSC-70 loading control and values for OP50 control were adjusted to 1 in order to calculate fold of change. For each condition, three independent replicates were used.

### Statistical analysis

All values are presented as mean ± standard deviation (SD). The differences between the control and experimental groups were compared using Student’s t-test, while one-way ANOVA followed by Tukey post hoc test was used for multiple comparisons. The differences between survival curves in nematode killing assays were analyzed using the log-rank (Mantel-Cox) test. A p value less than 0.05 was considered statistically significant. The statistical analysis and graphs were done in GraphPad Prism version 8.0.0 for Mac, GraphPad Software, San Diego, California USA, www.graphpad.com.

## Supplementary Information

Supplementary Information.

## Data Availability

The datasets generated during and/or analyzed during the current study are available from the corresponding author on reasonable request.
