# Supplementary material for: Probiotic-mediated p38 MAPK immune signaling prolongs the survival of Caenorhabditis elegans exposed to pathogenic bacteria
Source: Sci Rep. 2021 Oct 28;11:21258. doi: 10.1038/s41598-021-00698-5 (PMC8553853; doi:10.1038/s41598-021-00698-5)
Supplement: Supplementary file 1 — Supplementary Information. [file 41598_2021_698_MOESM1_ESM.pdf]

Probiotic-mediated p38 MAPK immune signaling prolongs the survival of *Caenorhabditis elegans* exposed to pathogenic bacteria

Miroslav Dinić[1*], Stefan Jakovljević[1], Jelena Đokić[1], Nikola Popović[1], Dušan Radojević[1], Ivana Strahinić[1], Nataša Golić[1]

[1]Laboratory for Molecular Microbiology (LMM), Institute of Molecular Genetics and Genetic Engineering (IMGGE), University of Belgrade, Belgrade, Serbia

SUPPLEMENTARY FIGURES

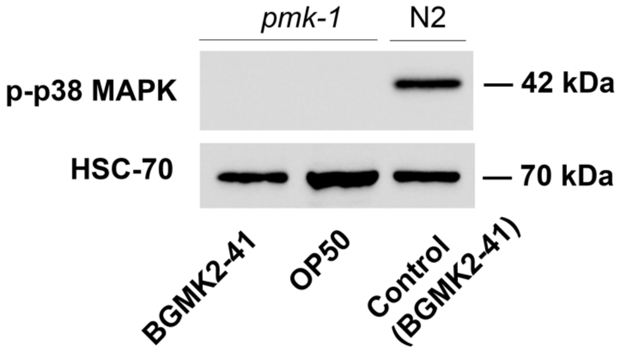

Supplementary Figure 1. Representative western blot confirming the defective p38 MAPK signaling in *pmk-1* mutant. WT animals treated overnight with heat-inactivated *Lactobacillus curvatus* BGMK2-41 was used as positive control. HSC-70 was used as loading control.

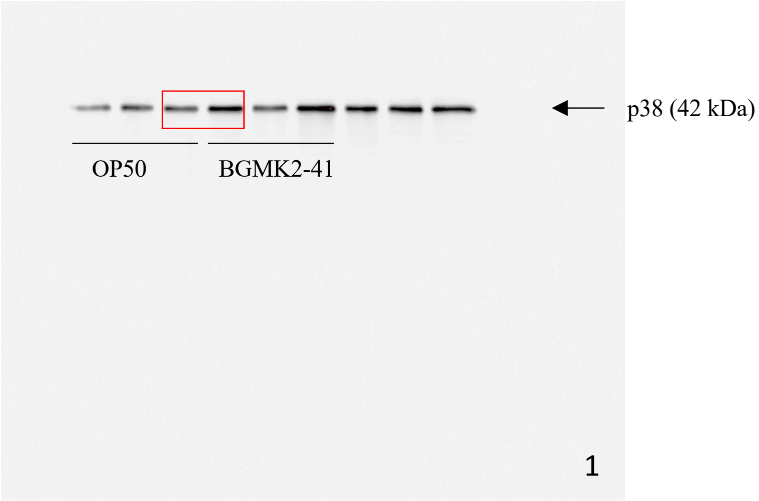
p38 (42 kDa)
OP50
BGMK2-41
1

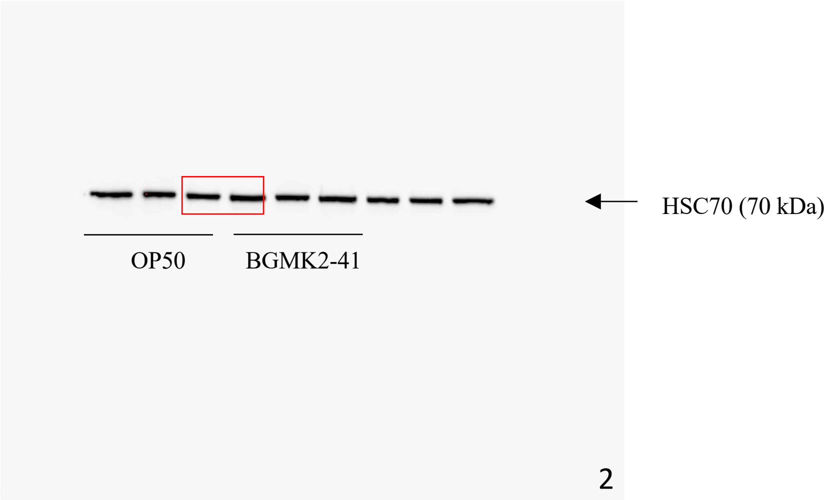
HSC70 (70 kDa)
OP50
BGMK2-41
2

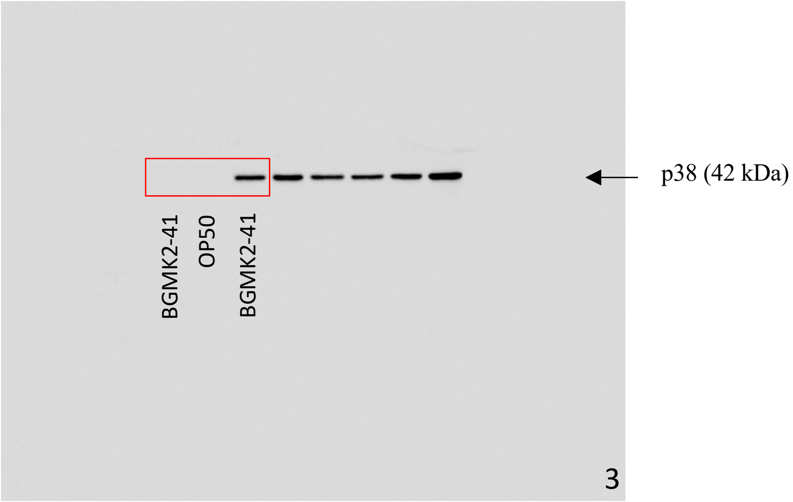
p38 (42 kDa)
BGMK2-41
OP50
BGMK2-41
3

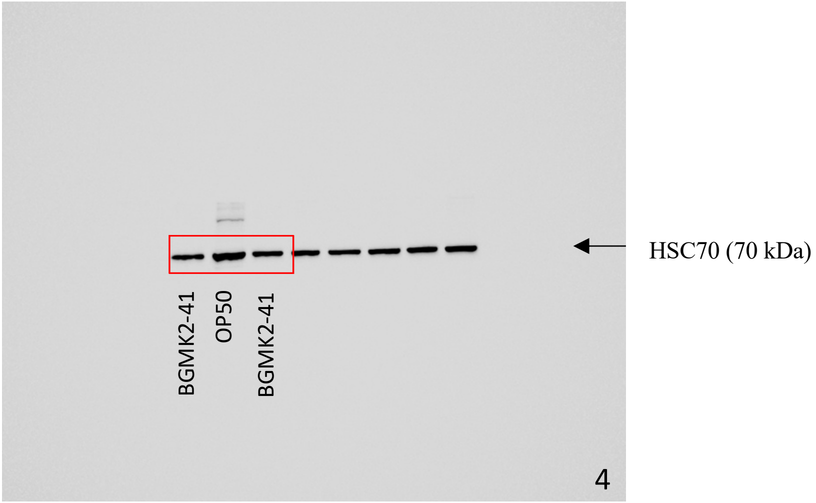

Supplementary Figure 2. Uncropped original images of Western blotting captured with ChemiDoc Touch Imaging System with Image Lab Touch Software (Bio Rad). The Image Resolution/Sensitivity scale was set to 4x4 pixel binning settings and pictures captured by using rapid auto exposure mode. Crop bands used in the **Figure 2** (1 and 2) and **Supplementary Figure 1** (3 and 4) are marked with red boxes.
